# Marked Hypoleptinemia Precedes Overt Fat Loss in Immune Checkpoint Inhibitor-induced Acquired Generalized Lipodystrophy

**DOI:** 10.1210/jcemcr/luad025

**Published:** 2023-03-09

**Authors:** Maheswaran Dhanasekaran, Rashi Sandooja, Alexandra S Higgins, Vinaya Simha

**Affiliations:** Department of Endocrinology, Diabetes and Nutrition, Mayo Clinic, Rochester, MN, USA; Department of Endocrinology, Diabetes and Nutrition, Mayo Clinic, Rochester, MN, USA; Department of Oncology, Mayo Clinic, Rochester, MN, USA; Department of Endocrinology, Diabetes and Nutrition, Mayo Clinic, Rochester, MN, USA

**Keywords:** hypoleptinemia, immune checkpoint inhibitor, lipodystrophy, pembrolizumab, panniculitis, adipocytes

## Abstract

Immune checkpoint inhibitors (ICIs) targeting cancer cells that evade immune T-cell regulation have revolutionized the treatment of metastatic carcinomas. Unfortunately, secondary endocrinopathies associated with ICI, including adrenal insufficiency, primary hypothyroidism, autoimmune diabetes, and rarely hypoparathyroidism, are increasing. Lipodystrophy, presumably due to the autoimmune destruction of adipocytes, leading to metabolic complications, is a less recognized adverse effect of ICI therapy. We present a case of a 66-year-old Caucasian woman treated with pembrolizumab, an anti-programmed death 1 inhibitor, for metastatic lung adenocarcinoma. Fifteen months after the treatment initiation, she was found to have hyperglycemia, hyperlipidemia, and hepatic steatosis but without any evidence of autoimmune diabetes. She was also noted to have isolated buccal fat pad loss, raising suspicion of acquired lipodystrophy. Despite well-preserved subcutaneous fat over the trunk and limbs, she had undetectable serum leptin levels. Whole-body fluorodeoxyglucose (FDG)-positron emission tomography scan showed diffuse mild FDG activity throughout the subcutaneous tissue, suggesting underlying inflammation. Over the next 3 months, she developed progressive fat loss leading to generalized lipodystrophy. Adipose tissue dysfunction, secondary to ICI-induced subclinical panniculitis, precedes overt fat loss and is characterized by hypoleptinemia and metabolic abnormalities.

Lymphocyte interaction with checkpoint signals such as cytotoxic T-lymphocyte-associated protein 4, programmed death 1 (PD-1), lymphocyte activation gene 3, and the cluster of differentiation 28, 27 regulates immune response and self-tolerance [1]. Monoclonal antibodies against these immune checkpoint proteins are widely used for cancer treatment. Evasion of self-tolerance of native antigens by upregulated immune systems leads to various autoimmune toxicities, including endocrinopathies, in up to 40% of patients treated with immune checkpoint inhibitors (ICIs) [2]. The most common endocrine glands affected are the thyroid (thyroiditis and hypothyroidism and, less frequently, hyperthyroidism) and pituitary (secondary adrenal insufficiency, hypophysitis, and panhypopituitarism). Rarely adrenal gland (primary adrenal insufficiency) and pancreatic β-cells (autoimmune diabetes similar to type 1 diabetes) can be involved [1]. The involvement of adipose tissue leading to acquired generalized lipodystrophy (AGL) is a rare complication that has been recently described (3-8).

In this paper, we describe a case of AGL secondary to the PD-1 antibody, pembrolizumab, and the natural course of the disease, which throws light on the critical role of normal adipose tissue function in maintaining glucose and lipid homeostasis.

## Case Presentation

A 66-year-old Caucasian woman with a 20-pack/year smoking history presented with progressively worsening headache, nausea, vertigo, and gait instability for 2 weeks. Further evaluation revealed adenocarcinoma of the lung with cerebellar and peritoneal metastasis. Ninety percent of the tumor cells were positive for PD-L1, and next-generation sequencing demonstrated a KRAS G13C mutation. Following surgical resection of the cerebellar mass and gamma knife treatment, she was started on adjuvant combination chemotherapy with pembrolizumab, pemetrexed, and carboplatin for 4 cycles, demonstrating good structural response, and then transitioned to pembrolizumab monotherapy 200 mg intravenous every 3 weeks as maintenance therapy.

**Figure 1.**
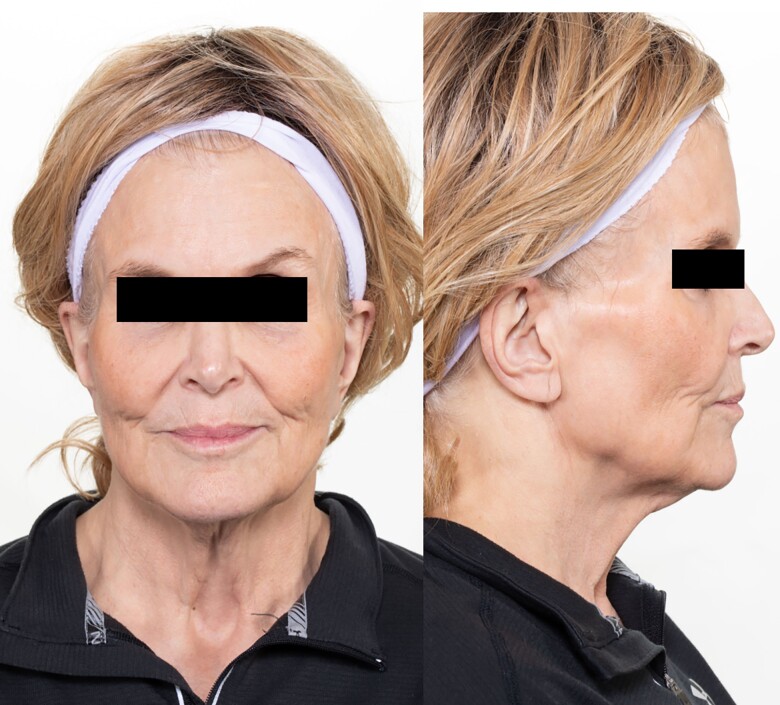
Buccal fat pad loss with the prominent zygomatic arch.

**Figure 2.**
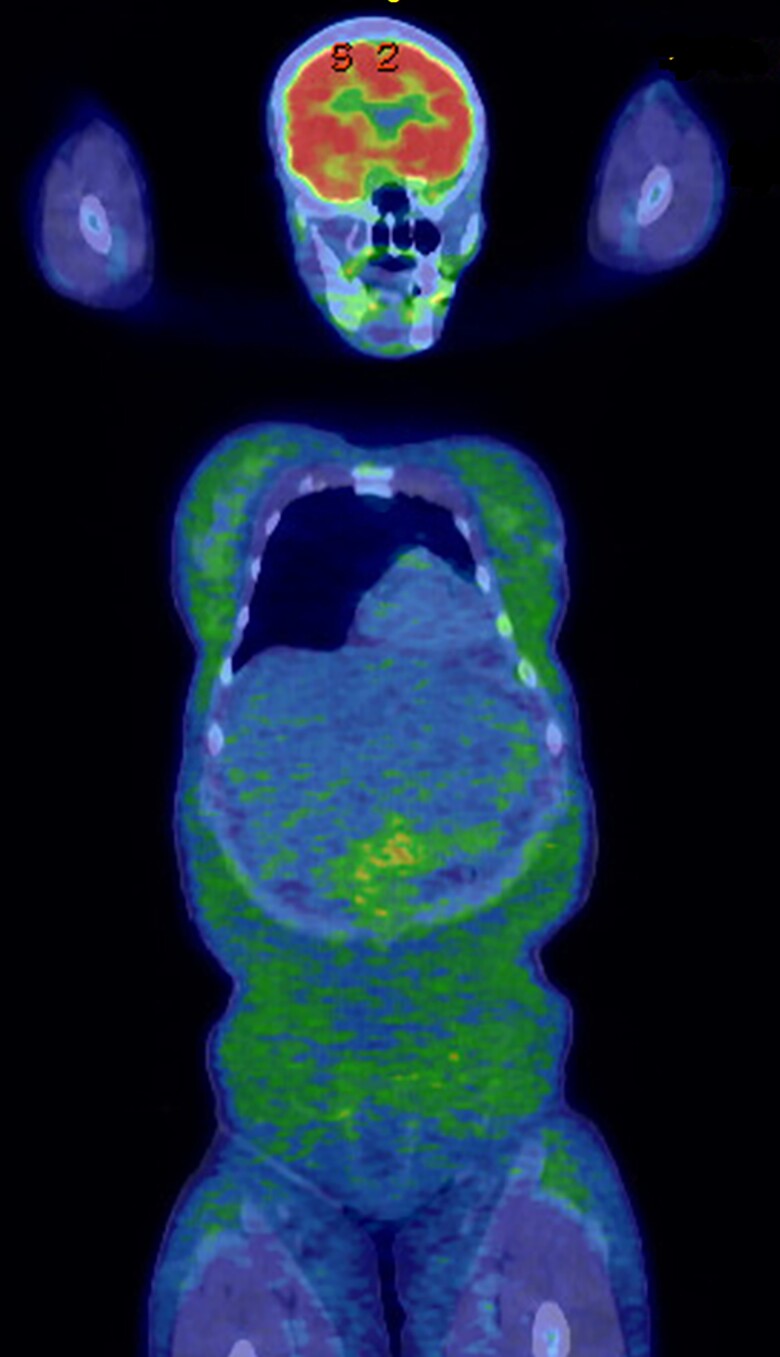
Whole body FDG-PET scan showing diffuse mild subcutaneous FDG activity suggestive of panniculitis. Abbreviations: FDG, fluorodeoxyglucose; PET, positron emission tomography.

**Figure 3.**
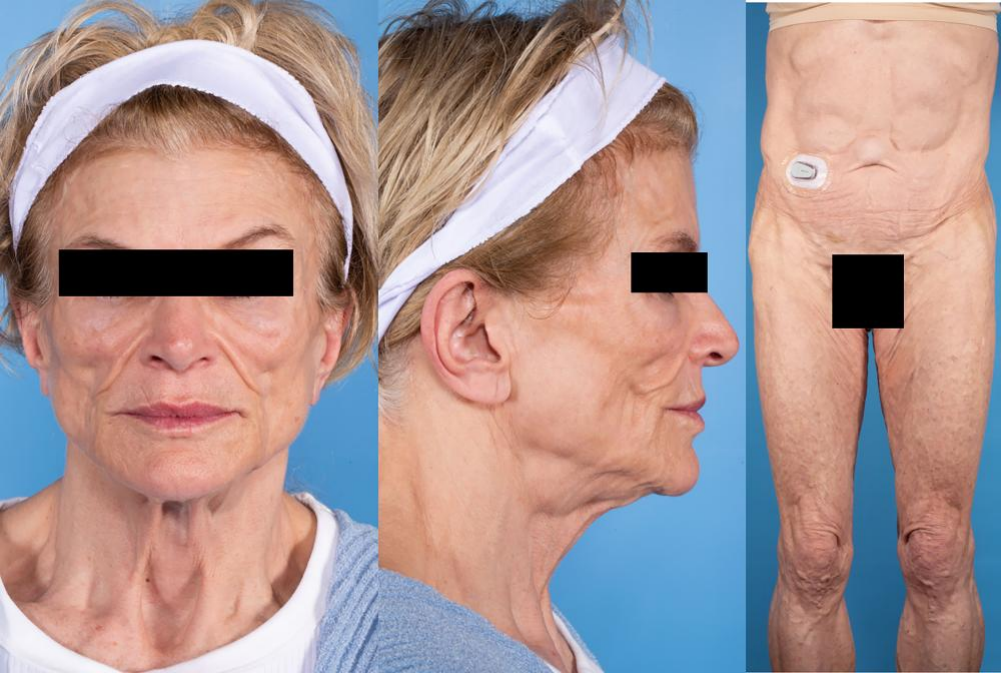
Three-month follow-up with generalized lipodystrophy.

Ten months after initiating pembrolizumab, she developed primary hypothyroidism and secondary adrenal insufficiency and was started on replacement therapy. Approximately 15 months following the treatment, she developed new-onset insulin-requiring diabetes mellitus without autoantibodies associated with type 1 diabetes. Prominent zygomatic arch with loss of buccal fat pad **(**Fig. 1**)** and hepatomegaly was noted clinically. Subcutaneous fat over the trunk and limbs was preserved, as evidenced by normal skinfold thickness measurements (Table 1). In addition to new-onset diabetes mellitus, she also developed new metabolic abnormalities, including hypertriglyceridemia of 436 mg/dL (4.93 mmol/L) [reference range: <150 mg/dL (<1.7 mmol/L)] and elevated liver enzymes (3-4 times the upper limit of normal). Subsequent liver biopsy revealed severe macrovesicular steatosis without any evidence of steatohepatitis. Notably, her serum leptin concentration measured during the initial assessment was undetectable at <0.6 ng/mL (reference range: 3.3-18.3 ng/mL) despite preserved fat in the trunk and limbs at this time. Whole-body fluorodeoxyglucose (FDG)-positron emission tomography (PET) scan obtained for cancer surveillance showed mild diffuse FDG activity throughout the subcutaneous tissues suggestive of panniculitis (Fig. 2). However, clinically she had no suggestive signs of panniculitis. Over the next 3 months, she lost 20 kilograms of weight along with generalized subcutaneous fat loss craniocaudally (Figs. 1 and 3) and continued to have marked hypoleptinemia.

**Table 1.** Skinfold thickness measured serially at different body sites demonstrating progressive loss of subcutaneous fat

	Chin	Biceps	Triceps (11-24)	Chest (7-19)	Abdomen (12-37)	Anterior thigh (20-40)
Mar 21	9 mm	4 mm	12 mm	11 mm	30 mm	28 mm
Apr 21	4 mm	3 mm	6 mm	6 mm	14 mm	12 mm
Jun 21	3 mm	3 mm	3 mm	(n/a)^a^	12 mm	12 mm

Numbers in parenthesis show 10th to 90th percentile values in normal population.

a Not available.

## Treatment

She was initiated on insulin therapy for new-onset diabetes and advised to follow a strict low-fat diet (less than 10-20 g fat per day). Pioglitazone 30 mg daily was trialed without any beneficial effect on fat loss and was ultimately discontinued after 4 weeks due to pedal edema.

## Outcome and Follow-up

Satisfactory control of metabolic abnormalities was achieved with diet and insulin therapy [moderate insulin requirement (0.6-0.8 unit/kg/day)]. Hemoglobin A1c has consistently been below 7% and serum triglycerides in the 200 to 250 mg/dL range with normal hepatic transaminase levels. In view of good metabolic control, she was not started on leptin replacement therapy despite generalized lipodystrophy and hypoleptinemia. She does have thrombocytopenia and splenomegaly, likely due to portal hypertension. She remains off systemic therapy for the malignancy with no evidence of disease progression in 12 months.

## Discussion

ICI-induced endocrinopathies involving the thyroid, pituitary, and rarely adrenal gland have been well documented in the literature [1]. However, ICI-induced autoimmune destruction of the adipocytes is a rare complication that is not fully understood. Lipodystrophy is characterized by partial or generalized subcutaneous fat loss, leading to metabolic complications, including insulin resistance, diabetes mellitus, hypertriglyceridemia, and hepatic steatosis [9]. AGL can be panniculitis associated (∼25%), autoimmune mediated (∼25%), or idiopathic (50%) [9]. The first case of ICI-induced AGL was reported in 2019 in a patient with advanced renal cell carcinoma on nivolumab therapy [3]. To date, 6 cases of ICI-induced AGL have been reported, all of whom were treated with anti-PD-1 inhibitors (4 cases with nivolumab and 2 cases with pembrolizumab) [3-8]. The clinical features and metabolic abnormalities noted in our patient are similar to the previously reported cases. Interestingly, our patient had an undetectable serum leptin concentration early in the disease course despite “near-normal” amounts of subcutaneous fat mass over the trunk and limbs. This suggests a functional defect in adipocytes that likely precedes overt fat loss. Diffuse increase in FDG-PET activity also supports the presence of subclinical inflammation. While no overt signs of panniculitis-like redness, tenderness, or nodule formation were noted, there was ongoing silent panniculitis causing metabolic abnormalities related to insulin resistance. Adipose tissue dysfunction, including defective adipokine secretion, can also cause metabolic disturbances associated with lipodystrophy and fat loss [10]. Severe hypoleptinemia has been reported in some of the previous case reports of ICI-induced AGL [4, 6], though leptin levels were not reduced in others [3, 5, 7]. This could be due to variations in the severity and extent of adipose tissue dysfunction and loss.

Treatment of AGL currently aims at controlling the metabolic sequelae of lipodystrophy, including insulin resistance and associated diabetes mellitus, hypertriglyceridemia, and nonalcoholic fatty liver disease/nonalcoholic steatohepatitis [10]. Our patient had reasonable glycemic control and normalization of serum transaminitis with a strict low-fat diet and insulin therapy. Leptin replacement therapy in generalized lipodystrophy, both congenital and acquired, has been shown to ameliorate metabolic abnormalities and is an approved treatment modality in these patients [10]. Whether it is safe and effective in ICI-induced AGL also needs to be determined.

Although rare, ICI-induced lipodystrophy has the potential to cause significant morbidity and long-term consequences, including atherosclerotic heart disease, pancreatitis, and liver cirrhosis. Early recognition and prompt treatment may help avoid this. Efforts to overcome weight loss through excess calorie consumption can potentially worsen metabolic abnormalities [10]. Hypoleptinemia and subclinical panniculitis may be important clues to the early detection of this condition.

## Conclusion

ICIs are currently approved for over 50 different types of cancers. Better understanding and risk stratification of immune-related adverse events are necessary to prevent fatal outcomes. A high index of clinical suspicion is critical as the symptoms are nonspecific, particularly in a catabolic milieu during cancer chemotherapy. A change in physical appearance, worsening glycemic control, or change in metabolic profile, including lipids or transaminitis, should raise the concern for possible lipodystrophy. Metabolic abnormalities may precede overt fat loss. Hypoleptinemia and diffuse FDG uptake of subcutaneous tissue suggest subclinical panniculitis and adipocyte dysfunction even before clinically evident lipodystrophy.

## Learning Points

- ICI-induced immune-related adverse effects, including secondary endocrinopathies, are increasing with wide application for different cancer types.
- Lipodystrophy, likely secondary to the autoimmune destruction of adipocytes leading to metabolic abnormalities, is a rare complication of ICI therapy.
- Clinical features of ICI-induced lipodystrophy, including fat loss, diabetes, dyslipidemia, and steatohepatitis, may be ascribed to underlying malignancy or other treatment effects, and a high index of clinical suspicion is warranted to make this diagnosis.
- Hypoleptinemia and evidence of subclinical panniculitis on FDG-PET may aid in early diagnosis.
- A low-fat diet and traditional glucose and lipid-lowering therapies can help control metabolic abnormalities. Leptin replacement therapy can be considered if these measures are not effective.

## Data Availability

Some or all data sets generated during and/or analyzed during the present study are not publicly available but are available from the corresponding author on reasonable request.
